# Effect of Hyperthyroidism on Bone Turnover Markers and Bone Mineral Density Before and After Treatment With Antithyroid Drugs

**DOI:** 10.7759/cureus.117092

**Published:** 2026-09-28

**Authors:** Bhabani Sankar Dhal, Padala Ravi Kumar, Deepak K Dash, Debasish Patro, Pradosh K Sahu

**Affiliations:** 1 Department of Endocrinology, Srirama Chandra Bhanja Medical College and Hospital, Cuttack, IND; 2 Department of Endocrinology, Maharaja Krishna Chandra Gajapati Medical College and Hospital, Berhampur, IND; 3 Department of Endocrinology, Diabetes, and Metabolism, Maharaja Krishna Chandra Gajapati Medical College and Hospital, Berhampur, IND

**Keywords:** bone mineral density, carbimazole, c-terminal cross-linked telopeptide, graves’ disease, procollagen type 1 n-propeptide

## Abstract

Background and objectives

Hyperthyroidism is a catabolic state with increased bone remodeling. Bone turnover markers (BTMs) and bone mineral density (BMD) may vary in patients with hyperthyroidism. The present study aimed to assess BTMs and BMD before and after antithyroid drug treatment in patients with hyperthyroidism.

Methods

This prospective observational study was conducted at a single tertiary care center from 4th November 2022 to 30th June 2024. Eighty-four newly diagnosed patients with hyperthyroidism, comprising men aged 18-65 years and premenopausal women aged >18 years, were enrolled. Of the 84 enrolled patients, 67 completed the follow-up and were analyzed. Sixty-five age-, sex-, and BMI-matched euthyroid subjects served as controls. BTMs, including procollagen type 1 N-propeptide (P1NP) and C-terminal cross-linked telopeptide (CTX), and BMD were assessed in all cases and controls. Carbimazole was initiated in all cases, and three months after achieving euthyroidism, BTMs were reassessed. BMD was assessed after one year of achievement of euthyroidism.

Results

Out of 67 hyperthyroid patients, 51 (76%) had Graves’ disease, 11 (16%) had toxic multinodular goiter, and five (8%) had toxic adenoma. Serum P1NP and CTX levels were significantly higher in cases compared to controls (P-value < 0.001 for both). BMD at the lumbar spine and neck of femur (NOF) was significantly lower in cases (P- value < 0.001 for all comparisons). Following carbimazole treatment, the median paired changes in P1NP and CTX levels were −143 ng/mL and −0.71 ng/mL, respectively. BMD increased by 10.18% at the lumbar spine, 12.42% at the right NOF, and 11.09% at the left NOF.

Conclusions

Patients with hyperthyroidism exhibited significantly lower BMD with increased BTMs. Treatment with carbimazole was associated with a decrease in BTMs and incomplete recovery of BMD at one year of euthyroidism.

## Introduction

Hyperthyroidism is a catabolic state with increased bone remodeling. Thyroid hormones stimulate osteoblasts to produce type 1 collagen and indirectly activate osteoclasts through receptor activator of nuclear factor kappa-Β ligand (RANKL), resulting in bone resorption, in addition to increased sympathetic activity and cytokine-induced bone loss [1-3].

Bone mineral density (BMD) and bone turnover markers (BTMs) are traditionally employed in assessing bone health. Studies on BMD in patients with Graves’ disease (GD) and toxic multinodular goiter (TMNG) have shown that BMD may be decreased or may remain normal [4-7]. The effect of antithyroid drugs on the recovery of bone mass and BMD has not been shown conclusively in the previous studies [6,8]. BTMs such as procollagen type 1 N-propeptide (P1NP) and C-terminal cross-linked telopeptide (CTX) in patients with hyperthyroidism have been reported to be increased or remain normal [9-11]. Moreover, the effect of antithyroid drugs on BTMs has been studied at variable time periods after initiation of antithyroid drugs [10,12]. A key gap in the existing literature is the lack of longitudinal data evaluating changes in both BTMs and BMD in a vitamin D-replete South Asian population, using a matched euthyroid control group and a standardized treatment approach. The present study aimed to assess changes in BTMs and BMD following carbimazole therapy in newly diagnosed patients with hyperthyroidism.

## Materials and methods

The study was carried out in the department of endocrinology of a tertiary care center from 4th November 2022 to 30th June 2024. This was a prospective observational study with baseline comparison between cases and controls and longitudinal follow-up of cases. The present study included men aged between 18 and 65 years and premenopausal women aged more than 18 years with newly detected hyperthyroidism. The baseline recruitment was completed in the first three months of the study and the last date of enrollment was 3rd February 2023. Institutional ethics committee approval (Institutional Ethics Committee, M.K.C.G Medical College, No. 1489; dated 03.11.2022) was obtained, and written informed consent was obtained from all study participants. The study procedures adhered to the guidelines of the Declaration of Helsinki 1964 and its subsequent revisions.

As the primary outcome in the present study was evaluation of treatment-related changes over time within the same subjects, sample size estimation was based on the paired t-test formula as follows:

$n = \{(Z_{1-\alpha/2} + Z_{1-\beta})^2 \times SDd^2\} / \Delta^2$

Where Z_1-α/2_ = 1.96 (for α = 0.05), Z_1-β_ = 0.84 (for power = 0.80), SD_d _= standard deviation of the differences between pairs, and Δ = expected mean difference.

The sample size was calculated based on the change in femoral neck BMD from a previous study by Dhanwal et al. [13] and was found to be 39. Taking the SD_d_ as 126.8 mg/cm^2^ and Δ as 57.0 mg/cm^2^. After adjusting for 20% attrition, the adjusted sample size was calculated to be 50. In the present study, 84 hyperthyroid patients were enrolled, of whom 67 completed the follow-up and were included in the per-protocol-based longitudinal analysis.

The hyperthyroid patients included GD, TMNG, and toxic adenoma (TA). The control group comprised healthy, euthyroid individuals recruited from nursing students and accompanying persons visiting the outpatient department (OPD) of the department of endocrinology. They were matched with the hyperthyroid cases based on age, sex, and BMI. A frequency-matching strategy was used during the recruitment process to ensure a comparable distribution of age, sex, and BMI between cases and controls. Patients with a diagnosis of subacute thyroiditis, diabetes mellitus, Cushing syndrome, chronic kidney disease, hypogonadism, metabolic bone diseases (MBD) such as parathyroid disorders, calcium/phosphorus metabolism disorders, and renal tubular acidosis (RTA) were excluded from the study. Besides pregnant women, smokers/alcoholics, or patients on medications like steroids, bisphosphonates, oral contraceptive pills, anti-cancer drugs, anticonvulsants, heparin, and lithium were also excluded. Patients with vitamin D deficiency (<20 ng/mL) were also excluded from the study. GD was diagnosed based on clinical and biochemical thyrotoxicosis with thyroid-stimulating hormone (TSH) receptor antibody (TRAb) positivity. TMNG and TA were diagnosed based on clinical and biochemical thyrotoxicosis with suggestive features on technetium-99m scan.

In all these patients, detailed history and anthropometric data (height, weight, BMI) were taken. Blood samples were collected in fasting state for estimation of complete blood count, renal/liver function tests, plasma glucose, glycated hemoglobin (HbA1c), calcium, phosphorus, alkaline phosphatase, free tri-iodothyronine (FT3), free thyroxine (FT4), TSH, and vitamin D (25(OH)D). A separate 4-mL EDTA (ethylenediaminetetraacetic acid) blood sample was collected in the morning after overnight fasting, centrifuged at 2000 rpm immediately, and preserved at a temperature of −80°C for estimation of serum P1NP and CTX. FT3, FT4, and TSH were estimated on a CLIA Siemens Advia Centaur CP machine (Siemens Healthineers, Erlangen, Germany) using standard kits supplied by the manufacturer. Serum TRAb levels were estimated by electrochemiluminescence immunoassay (ECLIA) using the Elecsys Anti-TSHR assay on a Roche Cobas e 411 Analyzer (Roche Diagnostics GmbH, Mannheim, Germany). Results were expressed in IU/L. A TRAb level >1.75 IU/L was considered positive. Serum P1NP and CTX were measured by electrochemiluminescence assay in a Roche Cobas e 411 Analyzer using total P1NP and CTX kits supplied by Roche Diagnostics following the manufacturer's instructions. The intra-assay coefficient of variation for CTX and P1NP was 2.1% and 2.7%, respectively. BMD was assessed at the lumbar spine (L1-L4) and right and left neck of femur (NOF) using the Hologic Discovery dual-energy X-ray absorptiometry (DEXA) scanner (Hologic Inc., Marlborough, MA). For quality control, the equipment was calibrated daily using the Hologic DXA spine phantom. The coefficient of variation (CV) was 0.392%.

All the patients were treated with an antithyroid drug using carbimazole and followed up at intervals of six to eight weeks, and the doses were adjusted based on thyroid function test (TFT) results. Definitive therapy was offered to patients with TMNG and TA, and as consent was not given, antithyroid drug was continued, and none of the patients received radioactive iodine therapy or surgery during follow-up. Routine calcium and vitamin D supplementation was not advised in any of the patients on follow-up. The euthyroid state was defined as normalization of FT3 and FT4 levels. After achieving euthyroid status for three months, serum P1NP and CTX were estimated, and after one year of achievement of euthyroidism with carbimazole therapy, BMD was measured in all the patients. Euthyroidism was maintained continuously through the 12 months of follow-up.

Statistical analysis

Normality of distribution was assessed using the Shapiro-Wilk test. Parametric data were expressed as mean ± standard deviation (SD), and comparison analysis was done using an independent t-test (inter-group), paired t-test (intra-group), and one-way ANOVA test (more than two groups). For comparisons among the three groups, post hoc pairwise analysis was performed using Tukey’s honestly significant difference (HSD) test. Non-parametric data were expressed as median (IQR), and comparison analysis was done using the Mann-Whitney U test (inter-group), Wilcoxon signed-rank test (intra-group), and Kruskal-Wallis test (more than two groups). Comparison between qualitative data was done using the chi-square test. Pearson's test (parametric) and Spearman's test (non-parametric) were conducted for correlation analysis. Least significant change (LSC) was calculated for BMD at different sites to look for statistically significant change with carbimazole treatment. Multivariable linear regression analysis was performed to identify independent determinants of change in BMD. A P-value < 0.050 was considered statistically significant. All data were analyzed using IBM SPSS version 29.0 (IBM Corp., Armonk, NY).

## Results

In the present study, 135 newly diagnosed hyperthyroid patients were screened, of whom 51 patients were excluded based on exclusion criteria (Figure 1). The remaining 84 hyperthyroid patients were included in the longitudinal follow-up study. From this, 17 patients were excluded (poor compliance, n = 12; lost to follow-up, n = 5), leaving a final cohort of 67 hyperthyroid cases. Out of 67 patients, 51 (76%) patients had GD, 11 (16%) patients had TMNG, and five (8%) patients had TA (Figure 1). A total of 75 healthy, euthyroid individuals were screened, of whom 10 (13.33%) were excluded for vitamin D deficiency. The remaining 65 (86.67%), matched with hyperthyroid cases for age, sex, and BMI, were enrolled as controls.

**Figure 1:**
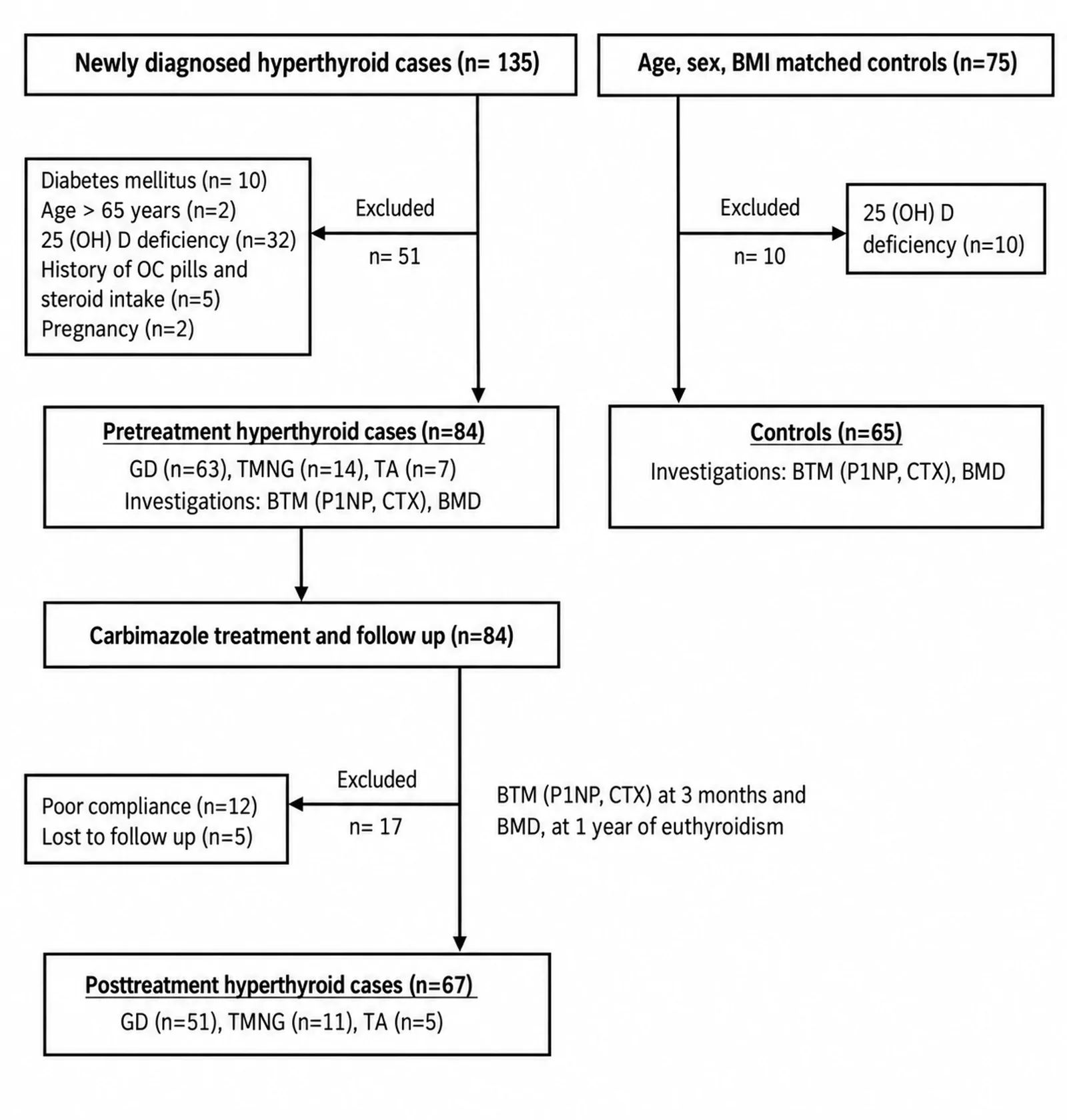
Flowchart depicting the study design and patient cohort. Abbreviations - 25(OH)D: 25-hydroxyvitamin D; BMI: body mass index; BMD: bone mineral density; BTM: bone turnover markers; CTX: C-telopeptide; GD: Graves’ disease; OC: oral contraceptive; P1NP: procollagen type I N-propeptide; TA: toxic adenoma; TMNG: toxic multinodular goiter.

Table 1 shows the baseline characteristics of cases and control groups. There was no significant difference between cases and controls for age, sex, BMI, and vitamin D levels. Four (5.97%) men in cases and three (4.62%) men in controls were above 50 years of age, whereas no women were above 50 years of age in either cases or controls. As expected, the FT3 and FT4 levels were higher, and TSH was lower in cases compared to controls. Serum P1NP (222.60 (147.30-324.90) vs. 36.51 (23.07-46.82) ng/mL) and CTX (1.41 (0.89-1.78) vs. 0.09 (0.06-0.14) ng/mL) levels were significantly elevated in hyperthyroid cases compared to controls. In Spearman’s correlation analysis, a significant positive correlation was found between P1NP and FT4 (r = 0.70, P < 0.001) and FT3 (r = 0.61, P < 0.001) levels. Similarly, CTX was found to be correlated significantly positively with both FT4 (r = 0.63, P < 0.001) and FT3 (r = 0.52, P < 0.001) levels. TRAb levels demonstrated a significant negative correlation with femoral neck BMD (left: r = - 0.32, p = 0.020; right: r = - 0.39, p = 0.005). Conversely, no statistically significant correlations were observed between TRAb levels and lumbar spine BMD (r = -0.21, p = 0.126), baseline P1NP (r = 0.15, p = 0.290), or CTX (r = 0.08, p = 0.580) levels. Abnormal Z-score (≤ -2.0) was present in 34 (50.75%), 32 (47.76%), and 32 (47.76%) of hyperthyroid patients at the spine (L1-L4), right NOF, and left NOF, respectively. Areal BMD at lumbar (L1-L4) spine (0.805 ± 0.121 vs. 0.997 ± 0.092 g/cm²) and at NOF (right: 0.636 ± 0.114 vs. 0.871 ± 0.110; left: 0.649 ± 0.106 vs. 0.877 ± 0.118 g/cm²) was significantly lower in cases compared to controls.

**Table 1:** Comparison of baseline characteristics in hyperthyroid cases and controls. Data are expressed as mean ± SD. * Data are expressed as median (interquartile range; 25 percentile-75th percentile). P: Comparison of variables between cases and controls in terms of mean and median. Abbreviations - SD: standard deviation; IQR: interquartile range; BMI: body mass index; FT3: free triiodothyronine; FT4: thyroxine; TSH: thyroid-stimulating hormone; 25(OH)D: 25-hydroxyvitamin D; P1NP: procollagen type I N-propeptide; CTX: C-telopeptide; Rt. NOF: right neck of femur; Lt. NOF: left neck of femur; L1-L4: lumbar vertebrae 1-4.

Variables	Cases (n = 67)	Control (n = 65)	P-value
Age (years)	35.25 ± 11.97	33.91 ± 10.23	0.490
Sex (M/F)	13/54	17/48	0.350
BMI (kg/m²)	21.73 ± 3.21	22.01 ± 2.35	0.569
TSH (µIU/mL)*	0.005 (0.00-0.16)	2.57 (1.48-3.48)	<0.001
FT4 (ng/dL)	6.01 ± 2.79	1.29 ± 0.26	<0.001
FT3 (pg/mL)	14.65 ± 4.45	3.28 ± 0.50	<0.001
25(OH)D (ng/mL)*	30.20 (25.80-34.90)	31.77 (29.41-34.34)	0.174
P1NP (ng/mL)*	222.60 (147.30-324.90)	36.51 (23.07-46.82)	<0.001
CTX (ng/mL)*	1.41 (0.89-1.78)	0.09 (0.06-0.14)	<0.001
L1-L4, Z-score	-2.20 ± 1.10	-0.41 ± 0.97	<0.001
L1-L4 (g/cm²)	0.805 ± 0.121	0.997 ± 0.092	<0.001
Rt. NOF, Z-score	-1.88 ± 0.94	-0.05 ± 0.94	<0.001
Rt. NOF (g/cm²)	0.636 ± 0.114	0.871 ± 0.110	<0.001
Lt. NOF, Z-score	-1.85 ± 0.88	-0.10 ± 0.93	<0.001
Lt. NOF (g/cm²)	0.649 ± 0.106	0.877 ± 0.118	<0.001

Among the various groups of hyperthyroid patients, there were no significant differences between GD, TMNG, and TA for age, sex, BMI, P1NP, CTX, and BMD at the right and left NOF. However, there was significantly lower BMD at the lumbar spine in TA patients compared to TMNG (0.660 ± 0.109 vs. 0.831 ± 0.101 g/cm²) and GD cases (0.660 ± 0.109 vs. 0.813 ± 0.113 g/cm²) (Table 2).

**Table 2:** Exploratory analysis of baseline characteristics between hyperthyroid subgroups. Data are expressed as mean ± SD. * Data are expressed as median (interquartile range; 25 percentile-75th percentile). # Comparison between GD vs. TMNG (P < 0.050). ## Comparison between TMNG vs. TA (P < 0.050). ### Comparison between GD vs. TA (P < 0.050). Abbreviations - GD: Graves’ disease; TMNG: toxic multinodular goiter; TA: toxic adenoma; BMI: body mass index; FT3: free triiodothyronine; FT4: thyroxine; TSH: thyroid-stimulating hormone; 25(OH)D: 25-hydroxyvitamin D; P1NP: procollagen type I N-propeptide; CTX: C-telopeptide; Rt. NOF: right neck of femur; Lt. NOF: left neck of femur; L1-L4: lumbar vertebrae 1-4.

Variables	GD (n = 51)	TMNG (n = 11)	TA (n = 5)	P-value
Age (years)	35.20 ± 11.50	32.91 ± 11.27	41.00 ± 18.39	0.462
Sex (M/F)	10/41	2/ 9	1/4	0.994
BMI (kg/m²)	21.76 ± 3.09	20.76 ± 3.96	23.62 ± 2.06	0.256
TSH (µIU/mL)*	0.00 (0.00-0.03)#	0.012 (0.007-0.050)	0.016 (0.007-0.0305)	0.012
FT4 (ng/dL)	6.03 ± 3.09	6.37 ± 1.34	5.04 ± 1.75	0.679
FT3 (pg/mL)	15.03 ± 4.47	13.78 ± 3.97	12.80 ± 5.48	0.445
25(OH)D (ng/mL)*	30.60 (26.30-36.40)	26.70 (24.50-30.00)	31.48 (22.63-36.14)	0.082
P1NP (ng/mL)*	222.60 (147.30-324.40)	198.10 (132.1-392.0)	257.40 (147.15-280.17)	0.886
CTX (ng/mL)*	1.44 (0.89-1.82)	1.49 (0.89-1.89)	1.00 (0.67-1.49)	0.746
L1-L4, Z-score	-2.10 ± 1.11	-1.90 ± 0.97##	-3.60 ± 1.20###	0.015
L1-L4 (g/cm²)	0.813 ± 0.113	0.831 ± 0.101##	0.660 ± 0.109###	0.019
Rt. NOF, Z-score	-1.77 ± 0.93	-2.20 ± 0.89	-2.34 ± 0.49	0.187
Rt. NOF (g/cm²)	0.649 ± 0.115	0.600 ± 0.097	0.591 ± 0.127	0.282
Lt. NOF, Z-score	-1.80 ± 0.89	-2.00 ± 0.73	-2.04 ± 0.65	0.780
Lt. NOF (g/cm²)	0.650 ± 0.114	0.628 ± 0.089	0.623 ± 0.127	0.676

The mean duration for achievement of euthyroidism after treatment with carbimazole was longer in GD (123 ± 15 days) than that of TMNG (105 ± 13 days) and TA (79 ± 10 days) patients.

After three months of euthyroidism achieved with carbimazole therapy, there was a significant reduction in the levels of P1NP and CTX, with median paired differences of -143 ng/ml and -0.71 ng/ml, respectively (Table 3). After one year of achievement of euthyroidism, abnormal Z-score (≤ -2.0) was present in 16 (23.88%), 17 (25.37%), and 14 (20.90%) of patients at the spine (L1-L4), right NOF, and left NOF, respectively. The areal BMD at lumbar spine significantly improved by 0.08 g/cm² (10.18%) and at NOF, right = 0.08 g/cm^2^ (12.42%) and left 0.07 g/cm² (11.09%), assessed after one year of euthyroidism, with 54 (80.6%), 46 (68.7%), and 47 (70.1%) individuals exceeding the LSC for lumbar spine (0.028 g/cm²), right NOF (0.042 g/cm²), and left NOF (0.044 g/cm²). No significant differences were found for the change in BMD at the NOF and BTMs across the three groups after antithyroid drug treatment (Table 4). In multivariable linear regression analysis, baseline BMD was a significant determinant of BMD change at the lumbar spine (β = -0.212, 95% CI: -0.303 to -0.120; P < 0.001) and left femoral neck (β = -0.224, 95% CI: -0.384 to -0.064; P = 0.006), whereas baseline FT4 levels were significantly associated with BMD change at the lumbar spine only (β = 0.007, 95% CI: 0.002-0.013; P = 0.008). No significant associations were observed for age, sex, disease etiology, weight change, baseline P1NP, or baseline CTX with BMD change at all sites.

**Table 3:** Comparison of parameters after treatment in hyperthyroid patients (n = 67). Data are expressed as mean ± SD. * Data are expressed as median (interquartile range; 25 percentile-75th percentile). Abbreviations - P1NP: procollagen type I N-propeptide; CTX: C-telopeptide; Rt. NOF: right neck of femur; Lt. NOF: left neck of femur; L1-L4: lumbar vertebrae 1-4.

After 3 months of normalization of thyroid hormones				
	Pre-treatment	Post-treatment	Δ (Delta)	P-value
P1NP (ng/mL)*	222.60 (147.30-324.90)	68.43 (57.38-101.47)	-143 (-235.11, -68.81)	<0.001
CTX (ng/mL)*	1.41 (0.89-1.78)	0.47 (0.27-0.65)	-0.71 (-1.34, -0.30)	<0.001
After 1 year of euthyroidism with antithyroid treatment				
L1-L4, Z-score	-2.20 ± 1.10	-1.40 ± 0.96	0.80 ± 0.42	<0.001
L1-L4 (g/cm²)	0.805 ± 0.121	0.887 ± 0.101	0.082 ± 0.050	<0.001
Rt. NOF, Z-score	-1.88 ± 0.94	-1.30 ± 0.82	0.580 ± 0.50	<0.001
Rt. NOF (g/cm²)	0.636 ± 0.114	0.715 ± 0.118	0.079 ± 0.073	<0.001
Lt. NOF, Z-score	-1.85 ± 0.88	-1.30 ± 0.87	0.580 ± 0.45	<0.001
Lt. NOF (g/cm²)	0.649 ± 0.106	0.721 ± 0.102	0.068 ± 0.060	<0.001

**Table 4:** Exploratory analysis of change in BTMs and BMD following treatment in hyperthyroid subgroups. Data are expressed as mean ± SD. * Data are expressed as median (interquartile range; 25 percentile-75th percentile). ## Comparison between TMNG vs. TA (P < 0.050). ### Comparison between GD vs. TA (P < 0.050). Abbreviations - BTMs: bone turnover markers; BMD: bone mineral density; GD: Graves’ disease; TMNG: toxic multinodular goiter; TA: toxic adenoma; P1NP: procollagen type I N-propeptide; CTX: C-telopeptide; Rt. NOF: right neck of femur; Lt. NOF: left neck of femur; L1-L4: lumbar vertebrae 1-4.

	GD (n = 51)	TMNG (n = 11)	TA (n = 5)	
Variables	Δ (Delta)	Δ (Delta)	Δ (Delta)	P-value
P1NP (ng/mL)*	-143 (-234.62, -74.91)	-145 (-290, -66.13)	-90.45 (-171.48, -74.81)	0.648
CTX (ng/mL)*	-0.85 (-1.40, -0.29)	-0.70 (-1.34, -0.31)	-0.60 (-0.74, -0.19)	0.373
L1-L4, Z-score	0.78 ± 0.43	0.65 ± 0.44##	1.32 ± 0.19###	0.016
L1-L4 (g/cm²)	0.075 ± 0.062	0.073 ± 0.065	0.145 ± 0.041	0.055
Rt. NOF, Z-score	0.55 ± 0.48	0.57 ± 0.38	0.92 ± 0.65	0.265
Rt. NOF (g/cm²)	0.070 ± 0.072	0.107 ± 0.062	0.106 ± 0.062	0.195
Lt. NOF, Z-score	0.57 ± 0.45	0.48 ± 0.55	0.90 ± 0.35	0.237
Lt. NOF (g/cm²)	0.061 ± 0.053	0.082 ± 0.070	0.106 ± 0.066	0.168

## Discussion

In the present study, we assessed the status of bone health in treatment-naive hyperthyroid patients. BTMs were found to be significantly increased, along with a decrease in BMD at the lumbar spine and NOF, in hyperthyroid cases. After treatment with antithyroid drugs, there were significant improvements in all these parameters at one year of euthyroidism, as marked by a reduction in BTMs with an increase in BMD.

In our study, both P1NP and CTX levels were significantly higher in hyperthyroid cases than in controls. Similar results have been reported in several previous studies [9,10]. This suggests that hyperthyroid patients have high bone turnover, leading to increased levels of both bone formation and resorption markers in serum. In the present study, both P1NP and CTX showed a strong positive correlation with FT3 and FT4 levels in hyperthyroid cases, similar to previous studies [5,10]. Higher levels of FT3 and FT4 lead to an increased rate of bone remodeling, leading to increased levels of turnover markers. The effect of thyroid hormones is mediated by the intracellular binding of T3 to the nuclear receptor (TR alpha) in osteoblasts [14]. It has been demonstrated that thyroid hormones stimulate osteoblasts to produce type 1 collagen and increase the expression of RANKL mRNA in osteoblasts, which activates RANK present in osteoclast precursors, resulting in bone resorption [1,14,15]. Increased cytokines and beta-adrenergic activity also contribute to bone resorption. The continuous process of bone remodeling through balanced resorption and deposition is essential to maintain the integrity and strength of the human skeleton. In the hyperthyroid state, the cycle occurs in roughly half the normal time, i.e., three to four months. This accelerated rate of bone turnover increases the ratio of resorption to bone formation, ultimately leading to net bone loss [16]. In the present study, BTMs were found to be decreased significantly at three months after achievement of euthyroidism and are consistent with the study by Singh et al., who observed normalization of BTMs within one to six months of antithyroid treatment [9]. Garnero et al. found normalization of BTMs within one month, suggesting early reversibility of BTMs with treatment [12]. These findings suggest that BTMs may help monitor the skeletal response to treatment with antithyroid drugs in hyperthyroid patients. But they have a limited role in predicting BMD changes, as shown in the present study.

In the present study, BMD was reduced significantly at lumbar spine and NOF in hyperthyroid cases, with about half of the patients below the expected range for their age. This has been reported by several authors previously [16-18]. Several authors have reported that cortical bone is more affected than trabecular bone in hyperthyroidism [19-21]. Bianchi et al. observed that in hyperthyroid patients, around a 40% increase in osteoclast resorption occurs in cortical bone, compared to only a 2.7% increase in trabecular bone [21]. In our study, BMD was significantly reduced at the lumbar spine as well as at NOF in hyperthyroid cases, suggesting involvement of both cortical and cancellous bone in hyperthyroid patients, concordant with previous studies [19,21]. In the present study, after one year of achievement of euthyroidism with antithyroid treatment, BMD was improved significantly across all sites in hyperthyroid cases. Similarly, Dhanwal et al. found a significant improvement in BMD at the lumbar spine, hip, and NOF after one year of antithyroid treatment [13]. There was a 10.18%, 12.42%, and 11.09% increase in the mean areal BMD at L1-L4 vertebrae, right NOF, and left NOF, respectively, after one year of achievement of euthyroidism, and these increases were greater than LSCs for areal BMD at all the respective sites, suggesting a significant increase with carbimazole treatment. Studies regarding the reversibility of bone loss after thyroid function normalization with treatment have found variable results in hyperthyroid patients. Krølner et al. in their study observed only a 5% increase in BMD after a two-year treatment period, whereas Rosen et al. demonstrated an 11% lumbar bone density increase after thyroid function normalization [22,23]. In contrast, Toh et al. demonstrated insignificant recovery of BMD after euthyroid status [24]. After successful treatment, BMD often returns to a normal level but requires three to six years of euthyroid state [6,19,25]. This might be a possible explanation for the partial recovery of BMD after short-term treatment, and probably because of the differential action of thyroid hormones on cortical and trabecular bone. In the current study, absolute BMD gains were similar at the lumbar spine and femoral neck, though percentage gains were numerically greater at the femoral neck. Overall, the observations of this study are in agreement with the majority of previous studies, which found that BMD partially recovered after one year of achieving euthyroid status [6,22,23,26].

In the present study, we have assessed BTMs and BMD comprehensively in a good number of hyperthyroid patients encompassing GD, TMNG, and TA patients. However, this study has a few limitations. This is a single-center study with a per-protocol analysis restricted to adherent completers, and controls were not followed longitudinally. The duration of follow-up is only one year after achieving euthyroid status. We have not assessed BMD at the distal end of the radius. The results of subgroup analysis could not be externally validated because of the small sample size in TMNG and TA groups. The effect of other modalities of treatment (radioiodine ablation and surgery) could not be assessed. Exclusion of postmenopausal women, older men, and vitamin D-deficient patients limits the generalizability of the study.

## Conclusions

Hyperthyroidism significantly accelerates bone turnover and decreases BMD at both the lumbar spine and femoral neck. Restoration of euthyroidism with antithyroid treatment rapidly suppresses BTMs, with significant improvement but incomplete recovery in BMD at one year, indicating that the detrimental skeletal effects of hyperthyroidism may persist even after biochemical euthyroidism. These findings highlight the importance of timely diagnosis and treatment of hyperthyroid cases and suggest that skeletal assessment may be considered in selected patients at higher fracture risk. Whether routine monitoring improves outcomes requires further study.
